# Linking intercontinental biogeographic events to decipher how European vineyards escaped Pierce’s disease

**DOI:** 10.1098/rspb.2024.1130

**Published:** 2024-10-02

**Authors:** Eduardo Moralejo, Àlex Giménez-Romero, Manuel A. Matías

**Affiliations:** ^1^ Tragsa, Passatge Cala Figuera, no. 6, Mallorca, Balearic Islands, Palma de Mallorca 07009, Spain; ^2^ Instituto de Física Interdisciplinar y Sistemas Complejos IFISC (CSIC-UIB), Campus UIB, Palma de Mallorca 07122, Spain

**Keywords:** *Xylella fastidiosa*, *Philaenus spumarius*, biological invasions, climate change, epidemic risk

## Abstract

Global change is believed to be a major driver of the emergence of invasive pathogens. Yet, there are few documented examples that illustrate the processes that hinder or trigger their geographic spread. Here, we present phylogenetic, epidemiological and historical evidence to explain how European vineyards escaped *Xylella fastidiosa* (Xf), the vector-borne bacterium responsible for Pierce’s disease (PD). Using Bayesian temporal reconstruction, we show that the export of American grapevines to France as rootstocks to combat phylloxera (~1872–1895) preceded the spread of the Xf grapevine lineage in the USA. We found that the time of the most recent common ancestor in California dates to around 1875, which agrees with the emergence of the first PD outbreak and the expansion into the southeastern US around 1895. We also show that between 1870 and 1990, climatic conditions in continental Europe were mostly below the threshold for the development of PD epidemics. However, our model indicates an inadvertent expansion of risk in southern Europe since the 1990s, which is accelerating with global warming. Our temporal approach identifies the biogeographical conditions that have so far prevented PD in southern European wine-producing areas and predicts that disease risk will increase substantially with increasing temperatures.

## 1. Introduction

No crop better illustrates the dangers associated with global plant trade than grapevine [1]. During the 19th century, the accidental introduction of powdery mildew (*Uncinula necator*) [2] into Europe ~1845 from exported North American wild *Vitis* species and the subsequent search for resistant germplasm preceded the arrival of other native pests and pathogens from the American continent—e.g. phylloxera (*Daktulosphaira vitifoliae*) ~1866 [3] and downy mildew (*Plasmopara viticola*) ~1878 [4]. In less than two decades, the entire European wine industry was devastated by the phylloxera crisis, while downy and powdery mildew have since become endemic diseases. These events coincide with the acceleration of human activity and the onset of global change.

The global spread of American grapevine pathogens and pests was triggered by the lack of proper sanitary control of plant propagating material, coupled with improvements in shipping and railroad transportation in the nineteenth century [5]. Surprisingly, the bacterium *Xylella fastidiosa* (Xf) subsp. *fastidiosa*, the causal agent of Pierce’s disease (PD) and a limiting factor for viticulture in the southeastern US, was not exported to Europe. This absence is even more puzzling because Xf is a vascular pathogen that is commonly transmitted by xylem sap-feeding insects but can also be transmitted by grafting [6], as are many viruses introduced by this route [7]. It was initially speculated that PD non-presence might be owing to a lack of competent vectors [8] and/or to non-conducive climate conditions [9], but no compelling evidence has yet been provided to support this latter hypothesis.

PD was first described in 1884 in the vineyards of Anaheim, California. In less than a decade, the disease had spread throughout southern California and leaped to the Napa Valley in northern California [10]. In his study, Newton Pierce documented in detail the unprecedented outbreak that ruined Southern California’s thriving viticulture. Although a grapevine ‘degeneration disease’ similar to PD had been described in Florida since around 1895 [11], the first unequivocal identification of PD in southeastern US was not made until 1951 [11]. Additionally, some aspects of the aetiology of the disease remained obscure for a long time; in 1942, it was demonstrated that the pathogen is transmitted by insects belonging to the sharpshooter leafhoppers (Hemiptera: Cicadellinae) and spittlebugs (Hemiptera: Cercopoidae) [12], whereas the bacterial nature of the disease remained unclear until 1978 [13].

During the 20th century, the endemic southeastern US origin hypothesis for grapevine-associated Xf strains prevailed owing to the greater resistance of native American grapevines compared to European *Vitis vinifera* cultivars [14]. However, early genetic analyses using multi-locus sequence typing revealed very little genetic variation among Xf subspecies *fastidiosa* isolates from grapevines across the USA, suggesting a recent introduction and spread as a clonal complex [15,16] (hereafter referred to as Xf_PD_). Shortly before, Xf_PD_ strains were also demonstrated to cause almond leaf scorch in California and to infect other hosts [17]. To date, Xf_PD_ has been found in Mexico [18], Costa Rica [19], Taiwan [20], Majorca, Balearic Islands, Spain [21] and Israel [22]. Recent phylogenetic analysis of the global population of Xf_PD_ on grapevine using whole genome sequences has shown that it consists of two major clades, referred here as the Western US Clonal Complex (WUCC) and the Eastern US Clonal Complex (EUCC). Both major clades show small genetic distances and are nested within the larger genetic diversity of Xf subsp. *fastidiosa* isolates from Central America, the most likely centre of origin of the subspecies *fastidiosa* [23–25].

PD has long been considered a potential threat to the European wine industry. Such concerns have increased following the first detection of Xf in Europe in 2013 as the causal agent of olive quick decline syndrome in Puglia, Italy [26]. Since then, several sequence types belonging to different Xf subspecies have been detected in Corsica and the Provence–Alpes–Côte d’Azur (PACA) region of France, the Balearic Islands, Alicante (Spain), Portugal and Tuscany [27]. Common to all these outbreaks is that the insect *Philaenus spumarius* plays a key role in the transmission of Xf-associated diseases in Europe, including PD [28,29]. Today, the three subspecies of Xf, *fastidiosa*, *multiplex* and *pauca*, occupy a small area of their potential geographical distribution in Europe according to recently published species distribution models (SDMs) [30,31]. However, these predictions disregard both epidemiological dynamics and vector distribution as limiting factors of invasiveness. A much more restricted potential distribution of PD has recently been proposed using mechanistic models [32].

Our understanding of the biology of invasive pathogens is biased towards successful events, while little attention has been paid to the underlying causes of failure in some emerging diseases. These unidentified causes are a risk in themselves, as some hidden processes may be rapidly unleashed by global change. Although the outline of the recent evolutionary history of Xf_PD_ is known [24], there is some controversy over how the PD pathogen has spread in the US over time. Unravelling the early events of Xf_PD_ spread in the southeastern USA may allow the linking of crucial biogeographical events with an understanding of the historical causes of PD absence in Europe. Here, we integrate recent advances in Xf_PD_ population genetics, phylodynamic and epidemiological modelling to investigate the biogeographic events that may have precluded the introduction, establishment and spread of PD in Europe. The dynamics of PD epidemic risk in Europe are considered from 1870 to 2023, an extensive timeframe to reflect how climatic conditions that have historically prevented the development of PD are rapidly shifting with climate change.

## 2. Results

### (a) The phylloxera crisis in European vineyards preceded the spread of Xf_PD_ in the USA

Between 1872 and 1900, over 14 million cuttings of American *Vitis* species were exported from the USA to France as resistant rootstocks [33]. To shed new light on whether this plant material carried Xf_PD_ infections, we extended the phylogenetic analysis of the global Xf_PD_ population *sensu lato* to include closely related genetic isolates from almond and other hosts in addition to grapevine (average nucleotide identity > 99.5%; electronic supplementary material, datasets S1 and S2). Both maximum likelihood (ML) and Bayesian analysis without priors confirmed that the world population of Xf_PD_ (*n* = 239) splits into two major clades: the western US clonal clade (WUCC), which includes isolates from California and Spain, and the eastern US clonal clade (EUCC), in which isolates from Taiwan are nested (electronic supplementary material, figure S1). However, in contrast to the study by Castillo *et al*. [24], the three Georgia isolates (16M2, 16M3 and XF51 CCPMI) were basal to the whole Californian clade and therefore included as part of the WUCC (electronic supplementary material, figure S1*a*,*b*).

To trace the time of the most recent common ancestor (tMRCA) of the global Xf_PD_ population, we used a tip-dating strategy and a strict-calibrated molecular clock to assign dates to internal nodes of each geographic clade and subclade that showed sufficient temporal signal, such as the Taiwan and southeastern US subclades (electronic supplementary material, figure S2). This information was then used to estimate internal node dates and their high posterior densities (HPDs) by including them as priors in the Bayesian analysis for the whole tree.

**Figure 1.**
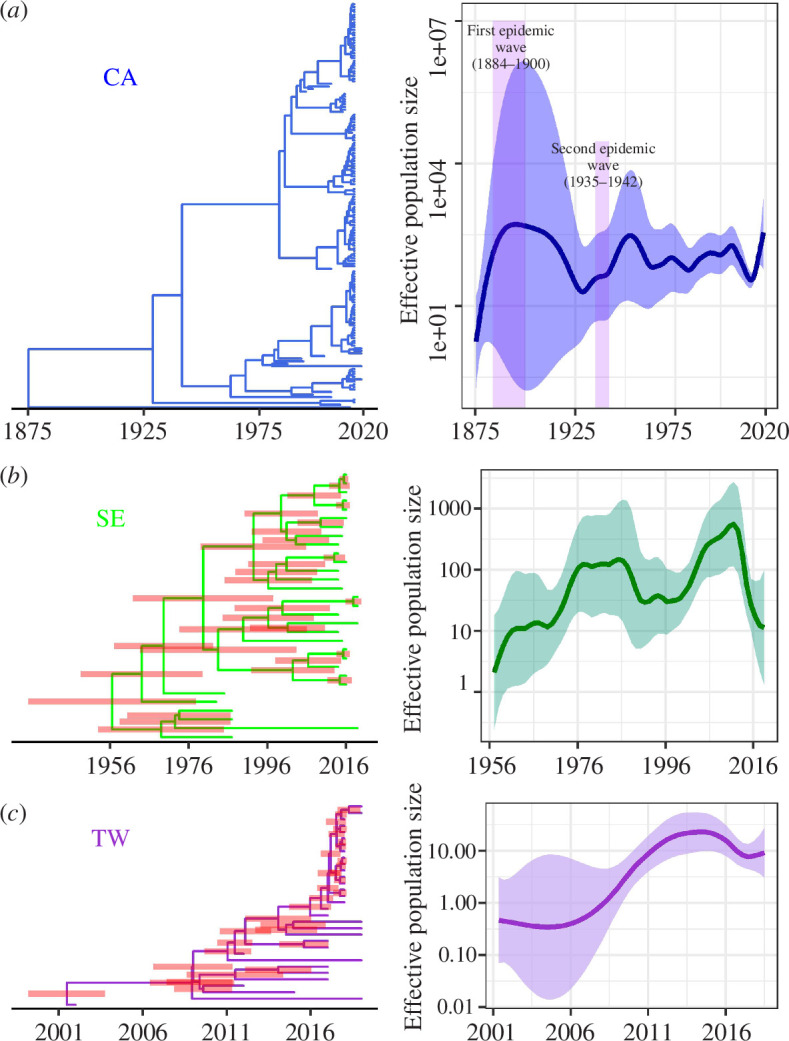
Regional expansion and emergence of PD epidemics. Temporal maximum clade credibility phylogenetic trees and their associated effective population size of PD epidemics in: California (CA) (*a*), southeastern US (SE) (*b*) and Taiwan (TW) (*c*). Isolate 14B1 from Georgia was included as an outgroup in the Californian subtree to show the divergence time between WUCC and EUCC. Node bars show 95% HPD. Purple columns represent the time of major reported PD epidemics in California.

**Figure 2.**
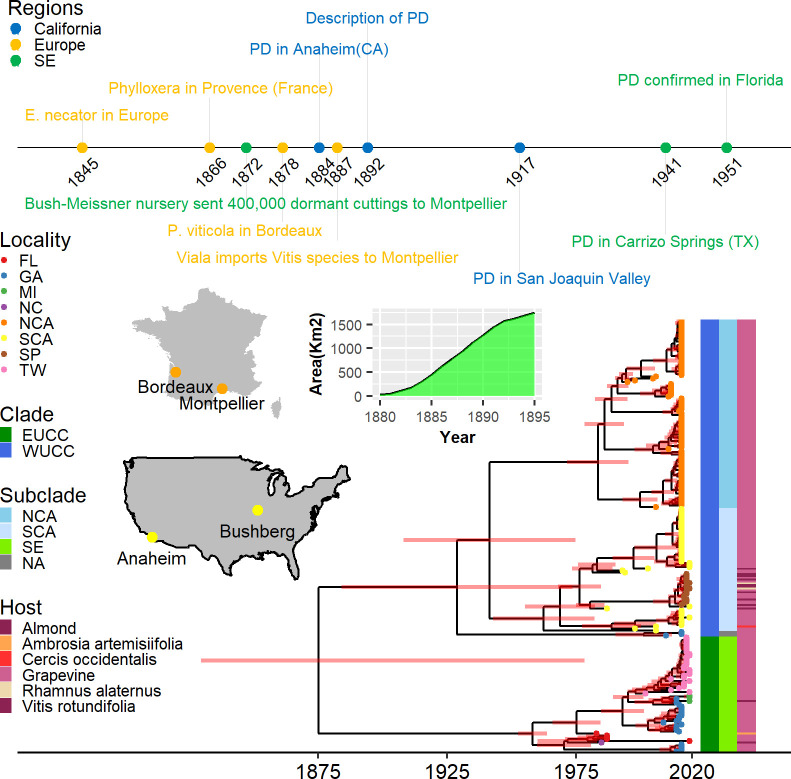
Time-calibrated maximum clade credibility phylogenetic tree of 239 Xf_PD_ genomes in the historical context of PD. Timeline of the major events related to vine diseases and export of plant material from USA to Europe. The area planted with American rootstocks in France increased significantly during the phylloxera epidemic. Most American vines were grown at Bushberg in Missouri and exported to Montpellier and Bordeaux concurrently with the first description of PD in Anaheim, California. These events align well with the estimated age of the tree, derived from the skyline demographic model and a log-normal relaxed clock. The divergence of the western US clonal clade (WUCC) and the eastern US clonal (EUCC) clades occurred at the time of the first epidemic around 1875 in southern California. Coloured bars show major clades, subclades and host; tree tips indicate the location of Xf_PD_ isolates. Abbreviations: Fl=Florida, GA=Georgia, MI= Mississippi, NC= North Carolina, NCA= North California, SCA= South California, SP=Spain,TX=Texas; NA= Not applicable.

Next, we took advantage of a previous study on the epidemiology of the almond leaf scorch outbreak in Majorca to estimate the tMRCA of the subclade using a logistic-growth tree prior [21]. A combination of a Hasegawa–Kishino–Yano (HKY) substitution model along with a strict molecular clock resulted in the best-fitting model, with a logistic growth rate coefficient (*k* = 0.65) very similar to that estimated for the almond leaf scorch epidemic (*k* ~ 0.6) [21]. In the extended phylogeny including isolates from *Rhamnus alaternus*, grapevines and almond trees, Xf_PD_ isolates from Majorca (*n* = 18) formed a monophyletic subclade nested within the WUCC and the tMRCA around 2005 (95% HPD; 1966–2015; electronic supplementary material, figure S3). All isolates from Taiwan (*n* = 31) clustered within the monophyletic EUCC clade (electronic supplementary material, figure S1 and figure 2) most likely originated from a single introduction event from the southeastern US before 2001 (95% HPD; 1997–2002; electronic supplementary material, table S1). An HKY-strict clock model combined with the Bayesian skyline coalescent model yielded the best-fitting phylogeny for the EUCC clade, with the tMRCA of the 32 isolates converging around 1956 (95% HPD; 1929–1972; figure 1).

Bayesian phylogenetic analyses using the above dated nodes as informative priors confirmed the early split of the original Xf_PD_ population into two deeply branched clonal clusters, as previously shown only for grapevine isolates [24]. The randomizing-date test revealed significant rates of evolution in the sampling times of bacterial genome sequences (electronic supplementary material, figure S4). Using the best-fit model (general time reversible (GTR)/uncorrelated log-normal (UCLN)/coalescent constant), we inferred a median molecular clock rate of 3.3 × 10^−7^ substitutions per site per year (95% HPD; 2.5 × 10^−7^– 4.2 × 10^−7^), which was similar across models and slightly lower than previous estimates [19,21]. Xf_PD_ isolates from almond and other hosts were clearly interspersed with grapevine isolates within their respective geographic subclades, and no migration within demes was detected (figure 2). The tMRCA of the northern Californian subclade was inferred to be around 1983 (95% HPD; 1969–1992) and that of the southern Californian subclade to be 1962 (95% HPD; 1937–1977), while the time of their divergence was estimated around 1941 (95% HPD; 1901–1967; figure 2). Notably, the long branches separating the WUCC and EUCC clades suggest a loss of representative samples from the Xf_PD_ ancestral populations and their replacement by a more recently adapted subpopulation [24]. In particular, the estimated tMRCA dates for both Californian subclades were substantially younger than the actual dates of the outbreaks reported by Newton Pierce in 1884 and the subsequent introduction into the Napa Valley around 1891 [10], indicating that the basal lineages to the WUCC are most likely extinct.

Thus, the current divergence of the Californian population likely arose after the second major PD epidemic wave (1935–1941) following replacement of the original Xf_PD_ population from the first epidemic wave (~1882–1900) [34] (figures 1*a* and 2). This would explain why, despite intensive sampling of Californian vineyards, no strains of this ancestral population have been detected to date [24]. It is also consistent with the theoretical results expected from periodic selection, whereby adapted ecotypes outcompete strains of the formerly dominant ecotype to their extinction [35]. The separation of WUCC and EUCC occurred soon after their introduction into the USA [25], as indicated in the models with the highest marginal likelihood estimates (electronic supplementary material, table S1). Although the direction of entry is unclear, the ancestral state reconstruction places 64.3% of trees in California as the site of introduction. Accordingly, the most parsimonious hypothesis is that some infected vines from the first epidemic wave in California were exported to the southeastern US (electronic supplementary material, figure S6). From this initial population in the southeastern US, only the EUCC lineage has survived to the present day, presumably in a process of geographic divergence and periodic selection of ecotypes more adapted to American grapevine varieties with some tolerance to PD. Conversely, Xf_PD_ isolates from Texas and Georgia, which are basal to the Californian clade, would indicate a second introduction from California to the southeastern US, starting with the second epidemic wave in California (1935–1941; posterior probability = 1; figure 1).

### (b) Phylodynamics support the timeline of reported epidemics

Based on the Xf_PD_ regional genealogies, we inferred the temporal effective population size of Xf_PD_ populations from Taiwan, the southeastern US and California using Skygrowth [36] (figure 1). The basic reproductive number *R*_0_ estimated from the temporal phylogenetic analysis shows reasonable agreement with those estimated from a temperature-driven epidemiological model [32] (electronic supplementary material, figure S6). In addition, we found a significant correlation between the growth rate of the number of infected vines inferred by Skygrowth and the risk index estimated by our epidemiological model for the southeastern US (Spearman’s rank correlation ρ = 0.310, *S* = 27 372, *p* = 0.01397) and southern California CA (ρ = 0.351, S = 27 029, *p* = 0.00476). These correlations, though weak, are remarkable given the extension of the study area. We also observed two major demographic peaks preceded by an increase in the effective reproductive number *R*_*t*_, consistent with the dates of the two major PD epidemic waves reported in California between 1884–1900 and 1935–1941 (figure 1*a*) [12]. The last epidemic wave (1935–1941 period) was much more extensive and more important in the Central Valley and coincided with the first detection of almond leaf scorch [37]. Such agreement between root height and PD epidemic cycles in California reinforces the accuracy of both the dated phylogeny and the temperature-driven epidemiological model.

### (c) PD epidemic risks in Europe from 1870 to present

Around 1872, soon after the discovery of phylloxera in the Rhône Valley [38], the first batches of imported American vines arrived in Montpellier (southeast France) and Bordeaux (Atlantic coast of France), mostly from the Bush & Son & Meissner nursery, Bushberg, Missouri, USA (figure 2). Despite the massive importation and propagation of American vines as rootstocks to combat phylloxera, there is no evidence that French vineyards were affected by PD during the nineteenth and twentieth centuries. To investigate whether this absence could be attributed to unsuitable climatic conditions, we assessed whether Xf_PD_ could become established and subsequently spread among vines if we forced the introduction of infected plants. We ran our epidemiological model to determine the epidemic risk of PD in Europe retroactively [32], using historical daily temperature projections from the Coupled Model Intercomparison Project fifth phase experiment (CMIP5) from 1850 to the present, and assuming a similar ecological range for the vector, *P. spumarius*, during this period. We found negative risk indices from 1850 to 1980 in most of continental France (>98% of the territory), with the only exceptions being the PACA region and the island of Corsica. In the PACA region, risk indices were mostly in the transitional risk category (−0.1 < *r* < 0.1) between 1872 and 1920 and in the very low or low risk category from 1921. In contrast, the risk index in Corsica remained low to moderate (electronic supplementary material, video S1).

**Figure 3.**
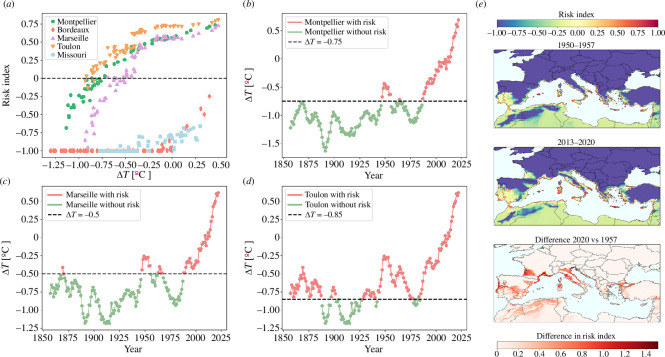
Potential PD epidemics in Europe through time. (*a*) The relationship between the risk index and the mean annual temperature anomalies for the French cities of Montpellier, Bordeaux, Marseille and Toulon and the American city of St Louis in Missouri. Data on temperature anomalies with respect to the 1991–2020 average for all locations between 1956 and 2023 were retrieved from the National Oceanic and Atmospheric Administration. Risk indices were estimated using hourly temperature data from the fifth generation of the European Centre for Medium-Range Weather Forescasts (ECMWF) atmospheric reanalysis for the global climate (ERA5-Land) applied to the model for the same period. (*b*) Time series from 1850 to the present showing temperature anomalies below and above the epidemic risk threshold for Montpellier (*b*), Marseille (*c*) and Toulon (*d*) derived from the relationship in (*a*). The epidemic risk is negative throughout the period 1850–2023 in Bordeaux (not shown). (*e*) Snapshots showing the evolution of PD risk maps for Europe from 1950–1957 to 2013–2020 and their differences. The risk index denotes the potential exponential growth of the epidemic; −0.10 to 0.10 transition risk, 0.10–0.33 low risk; 0.33–0.66 moderate risk and >0.66 high risk.

Owing to the low spatial resolution (~100 × 100 km grid) of the CMIP5 models used in this work, the epidemic risk might be underestimated at the local scale in hotter coastal areas and river valleys. To overcome this inconvenience, we searched for the relationship between the PD risk index and annual temperature anomalies (*ΔΤ*) for Montpellier, Bordeaux and the PACA region and the risk index derived from our model using the fifth generation of the European Centre for Medium-Range Weather Forecasts (ECMWF) atmospheric reanalysis for the global climate (ERA5-Land data;~10 × 10 km^2^ grid) from 1950 to the present. A strong non-linear correlation was observed, allowing a clear separation of *ΔT* threshold, below which the risk index would be expected to be negative (figure 3). Extending *ΔΤ* backwards in time to 1850, we found that risk indices for Bordeaux were negative from 1850 to the present, whereas risk indices for Montpellier were also negative (*ΔT <* −0.75) from 1850 to 1980, with a short positive 4-year period between 1949 and 1953 and an exponential increase in the risk index since the 1990s. In the PACA region, risk indices were positive most of the time, although there were continuous periods of negative risk (*r* < 0) from 1887 to 1896 and from 1902 to 1926, which were long enough to eliminate any incipient outbreak (figure 3).

Phylloxera, powdery mildew and downy mildew were introduced from the continent to the Mediterranean islands (Sicily, Crete, Sardinia, Balearic Islands, etc.) with infected vines at the end of the nineteenth century [39]. However, none of these plants carried X_fPD_ infections, despite our conclusion that PD epidemics could have occurred on the Mediterranean islands (*r* > 0) with little variation from 1850 to the present (electronic supplementary material, video S1 and figure S8). This finding suggests that in a hypothetical introduction of Xf_PD_ from American grapevines to France, latent infections in vines would last only a few years under climatic conditions unfavourable to the pathogen. Risk map projections using more reliable modern temperature data from 1950 to the present confirm moderate PD risks mainly in the Mediterranean islands and to a lesser extent locally in some continental Mediterranean coastal areas (figure 3*e* and electronic supplementary material, video S2). In addition, our model reveals temporal trends and ongoing epidemiological processes previously unidentified by correlative SDMs. Since the 1990s, there have been large shifts in the extent of PD risk zones and indices (i.e. *r* > 0) (electronic supplementary material, video S2), mainly within transitional and low-risk zones in Europe associated with a progressive increase in mean annual temperatures (figure 3*e*). Conversely, the Mediterranean islands with the highest risk indices showed low risk variation (s.e. = ± 0.1) over the same period (electronic supplementary material, figure S9). Over the past two decades, the largest increases from non-risk to transitional and/or low-risk zones occurred in important wine-growing areas located in the Douro, Garonne, Rhone and Po river basins (figure 3 and electronic supplementary material, video S2). These results agree with regional climate change trends for Europe [40], indicating that the model is sensitive enough to capture regional differences in climate velocity shifts in the risk index.

### (d) Risk of PD epidemics in American vines

Just as our model finds that temperatures in the late nineteenth century were probably unsuitable for PD outbreaks in most of continental Europe, temperatures in the American nurseries of origin (electronic supplementary material, video S1) were probably also unsuitable. Nearly all American wild vine germplasm exported to France to combat phylloxera was grown in a nursery at Bushberg, Missouri [38] (figure 2), well north of the *r* = 0 boundary in our current risk maps (electronic supplementary material, figure S12). This can be seen in electronic supplementary material, figure S13B, which shows the shrinkage of the PD risk area based on the thermal anomalies collected over the period (1957–2023). In our model, areas of PD risk in North America are significantly reduced in years with negative annual temperature anomalies, while in continental Europe the risk is almost zero in years with negative anomalies (electronic supplementary material, figure S13a).

American vine species and hybrids generally exhibit greater tolerance and/or resistance responses to Xf_PD_ than European *V. vinifera* varieties [14,41]. Consistent with this, we found that rootstocks derived from American vine hybrids developed significantly fewer severe PD symptoms and were less infected than rootstock/hybrid combinations with European varieties in the inoculation assays (electronic supplementary material, figure S10a). In addition, American rootstocks required approximately 1.3 times more accumulated modified growing-degree days (MGDD) to reach similar levels of symptom severity to European varieties within the range between 800 and 1027 MGDD (electronic supplementary material, figure S10b). To investigate the effect of climate on PD epidemics within wild *Vitis* populations, we tested our epidemic model by calibrating the average MGDD function from data obtained from American grapevine and hybrid inoculations. We obtained, as expected, a risk map for the eastern US that was very similar to the map calibrated with *V. vinifera*. This similarity is mainly because the average accumulated MGDD in summer in the eastern US is well above those required for PD epidemics (electronic supplementary material, figure S12 and video S3). Nonetheless, lower symptom development rates of American *Vitis* species could influence the survival of infections during winter. Even without considering the possible effect of winter curing, the limits of *r* > 0 in the PD risk map suggest that only populations of American wild vines inhabiting the coastal plains of the Gulf of Mexico (electronic supplementary material, video S3) could carry Xf_PD_ infections and that most plants obtained from US nurseries would have been free of Xf_PD_.

## 3. Discussion

The interacting effects of multiple global change drivers (e.g. temperature, agricultural intensification and trade in biological products) on outbreak risk of emergent pathogens have rarely been addressed. Our work provides new clues to understanding why PD has not become established in continental Europe, despite the massive importation of American vines during the nineteenth century [2–4]. To answer this question, we assessed the risk of developing PD outbreaks over time conditioned by the climatic suitability of European wine-producing regions in the event of the pathogen’s introduction (*cf*. [31]). We have also documented the chronology of the introduction and spread of Xf_PD_ in America and its subsequent introduction to other parts of the world. This approach provides a robust spatiotemporal framework to address potential epidemiological, environmental and historical factors that would explain why European vineyards have escaped PD.

The historical absence of PD in continental Europe could be attributed in decreasing order of robustness to four main factors: (i) the non-conductive climatic conditions for the establishment of Xf_PD_ and/or its main vector in the main European wine-producing areas (figure 3); (ii) the importation of American grapevines from the eastern US to Europe to combat phylloxera, which likely predated the introduction and spread of Xf_PD_ in the southeastern US (figure 2); (iii) the lower susceptibility and thus bacterial load of American vines and their hybrids compared to European *V. vinifera* varieties (electronic supplementary material, figure S10); and (iv) the unsuitable climate for PD in the nurseries of origin (US) at the time of export to France (electronic supplementary material, figure S12). Each of these factors could account for the exclusion of PD in the last quarter of the nineteenth century. Even when considering the temporal uncertainty of the dated phylogeny and epidemiological model, the combined likelihood of Xf_PD_ introduction, establishment and spread should be considered very low.

Climatic exclusion between 1870 and 1980 is strongly supported in our model. We highlight that the thermal dependence in the probability of developing PD symptoms was parameterized from inoculation experiments involving 36 *V. vinifera* varieties. The reliability of the model with the vector is high, and it predicts the current distribution of Xf subspecies *fastidiosa* in Europe as well as other subspecies [32]. PD risk maps based on reanalysis of ERA5-Land climate data from 1950 to the present, although initially built exclusively for Xf_PD_, also explain the recent developments of Xf-induced diseases in Europe (figure 3). This is well illustrated by the evolution of PD risk in the Mediterranean climate of southeastern France from 1956 to the present and Portugal, showing how the risk of establishment in these regions has only recently emerged (electronic supplementary material, videos S4 and S5).

Certainly, the non-interspersion of isolates from different countries within major phylogenetic clades suggests that phytosanitary controls on the international movement of grapevine germplasm have contributed to containing the spread of PD. To our knowledge, there has been no successful introduction of Xf_PD_ into Europe via infected grapevines. Although these facts would indicate effective controls, our PD risk maps strongly suggest that climate rather than phytosanitary measures played a greater role in the exclusion of Xf_PD_ from European vineyards during the twentieth century (figure 3). It appears that the current reduced distribution of Xf in Europe is not coincidental, occurring exclusively within the moderate PD risk zones (compare electronic supplementary material, figure S11 and video S1). This observation reinforces the predictions of our mechanistically based model. In future scenarios of +2 and +3°C annual temperature increases, we forecast that climate will no longer prevent potential outbreaks in Portugal, southern France and Italy [42]. Consequently, the prevention of Xf_PD_ in these areas will depend solely on the implementation of effective surveillance and phytosanitary measures.

The chronology of our dated phylogeny implies that American vines exported to France between 1875 and 1900 should have been free of Xf_PD_ infection (figure 2). Our Bayesian ancestral state reconstruction dates the tMRCA of the global Xf_PD_ population to 1875 (95% HPD: 1735–1941). Although the reconstruction gives a higher probability for a Californian origin, the results are still inconclusive owing to the lack of sampling of Xf_PD_, both close to the root of the phylogenetic tree [43] and in natural vegetation in the southeastern US, which could help to discard its origin from this region. The hypothesis that Xf_PD_ was introduced to southern California from Central America before 1880 and to the southeastern US from California soon after is more consistent with the data presented here and the timeline of reports of PD and ‘degeneration disease’ in California and the southeastern US, respectively. Although a southeastern US origin for Xf_PD_ is a plausible hypothesis, it is not supported by genetic data or disease reports. This hypothesis is largely based on the observation that native vines exhibit greater resistance/tolerance to PD than European *V. vinifera*, suggesting a possible coevolutionary process [14]. It is also grounded in the historical disease problems of *V. vinifera* in the southeastern US [11]. Nonetheless, the ‘grape degeneration’ of European *V. vinifera* plantations in the southeastern US was notoriously not mentioned in either the Grapevine Manual of 1883 [44] or Viala Scientific Mission Report [33], where other grapevine diseases and pests were described in detail. The historical failures of European variety plantations could be explained by the action of a large number of other indigenous pathogens and pests (downy mildew, powdery mildew, anthracnose, black rot, nematode-borne viruses, etc.) besides Xf_PD_ [11]. Conversely, a recent study has shown that American grapevine species from the arid regions of the western US and Mexico, where Xf_PD_ is not currently found, are more resistant to PD than vine species from the southeastern US and Mexico. These findings do not support the endemic hypothesis for Xf_PD_ [41].

In our inoculation trials, American rootstock hybrids required on average 1.3 times more cumulative *MGDD* to show similar levels of PD symptoms than European *V. vinifera* varieties (electronic supplementary material, figure S10). This lower susceptibility generally implies lower bacterial loads in infected vines, delays in symptom development, lower plant-to-plant transmission and a greater chance of winter curing at equivalent temporal settings. In our epidemiological model, Xf_PD_ transmission within natural populations of American *Vitis* spp. would be highly unlikely northward beyond the coastal plains of the Gulf of Mexico. Although these rough predictions in wild American *Vitis* populations have not yet been validated in the field, there is some circumstantial evidence to suggest that Xf_PD_ transmission is not occurring. We searched the most recent European Food Safety Authority (EFSA) host database [45] for confirmation of infection in wild American *Vitis* in its native habitat. Surprisingly, the only rare records are of wild vines in their natural habitat in proximity to vineyards [46] or explants grown in nurseries and exposed to natural conditions. However, there are no documented cases of infected plants in their natural habitat. This lack of infection in natural populations of wild *Vitis* spp. is also suggested by the non-detection of Xf_PD_ DNA by PCR analysis in the National Clonal Germplasm Repository field genebank in Davis, CA, which contains more than 68 samples of grapevine species from the southeastern US [47].

We conclude that the lack of spatiotemporal concurrence of factors leading to the establishment of PD would explain its historical absence from continental Europe. Nevertheless, the expansion of the risk zones for PD since the 1990s has been accompanied by rising temperatures, which have increased the probability of outbreaks in areas where they were previously considered highly unlikely. By combining a dated phylogeny of the worldwide Xf_PD_ population with PD risk modelling, we can better discriminate between temporally overlapping events, thereby focusing solely on the effect of climate on PD risk. This approach can be extended to other pathosystems, particularly vector-borne pathogens. Our broad spatiotemporal analysis of PD risk sheds light on the effects that the direction and intensity of climate change may have on the likelihood of outbreaks in southern Europe. An important finding of the study is the need for active surveillance in southern Europe, where the risk of PD will increase from the current very low or low risk indices to moderate risk in the > +2° scenarios [42]. Early detection of Xf_PD_, when the potential exponential growth of PD is low, enables eradication measures that would not otherwise be feasible when risk indices reach moderate or high levels.

## 4. Methods

### (a) Phylogenetic analysis of the Xf_PD_

A total of 242 *X. fastidiosa* subsp. *fastidiosa* genomes were included in this study. For Xf_PD_ phylogenetic analysis, we selected 239 isolates from different hosts sharing >99.5% average nucleotide identity scores in their whole-genome sequence using JSpeciesWS [48] (electronic supplementary material, dataset S1). Gff files of coding sequences from previously annotated assembled genomes in the work of Castillo *et al.* [24] (*n* = 182) were kindly provided by the authors. The other assembled genomes were retrieved from the NCBI nucleotide database (*n* = 19) or de novo assembled from sequence read archives (SRAs) (*n* = 41) using SPAdes v. 3.14.1 [49] and evaluated with QUAST v. 4.5 [50]. Xf genome contigs in multiFASTA format were individually reordered and aligned to the Temecula1 assembly (GCA_000007245.1) reference genome using Mauve’s contig mover function [51]. The Prokka pipeline was used to annotate the assembled and reordered genomes [52]. A core genome alignment of the PD-causing isolates plus the three Costa Rican isolates (non-PD) and one isolate collated from a coffee plant from Mexico (CFPB8073) was created using the -e (codon aware multisequence alignment of core genes) and -n (fast nucleotide alignment) flags in Roary [53]. The resulting core genome alignment was then used to build a ML tree with Randomized Axelerated Maximum Likelihood (RAxML) [54] (electronic supplementary material, figure S14). Gubbins v. 2.2.0 [55] was used to generate recombination-masked full-genome length and single nucleotide polymorphism (SNP)-only alignments using default parameters and a minimum of three base substitutions required to identify recombination. ML phylogenies were obtained from the recombination-purged alignments using IQ-tree v. 1.6.7.2 [56] under the determined best-fit nucleotide substitution model (GTR+F+G4, as determined by ModelFinder [57]) and ultrafast bootstrapping of 1000 replicates. Trees were visualized and annotated with ggtree [58].

A total of 665 variable sites were generated as an output file in Gubbins, representing the core genome SNP-only alignments. These were then used to estimate substitution rates, emergence times and divergence dates using Bayesian approaches as implemented in BEAST v. 1.10 [59]. BEAUti xml files were manually modified to specify the number of invariant sites (A: 344747; C: 380496; G: 419538; T: 373243) in the core genome. All possible combinations (*n* = 12) of two substitution models (GTR+HKY), molecular clocks (strict, uncorrelated relaxed exponential/lognormal molecular clock) and tree demographic models (constant size coalescent, exponential population growth and Bayesian skyline coalescent) were compared using Bayes factors (BF) based on marginal likelihood estimates by path sampling and stepping stone calculations in BEAST [59]. Each analysis initially comprised two independent runs of 10 million generations sampled in every 10 000 states, and then the five best models in log Bayes factor (BF) were selected for two independent runs of 100 million generations. Tree calibration was performed by assigning the date of isolation to each tip of the tree. The temporal signal of the heterochronous sequence data was explored by performing a linear regression analysis of root-to-tip distances versus sampling time using TempEst 1.5 [60]. A date randomization test was performed using TipDatingBeast [61] to further assess the genetic and temporal signal present in a dataset. Markov chain Monte Carlo (MCMC) convergence was assessed in Tracer v. 1.7.1 [62], ensuring effective sample sizes greater than 200. Log-Combiner v. 1.10.4 [59] was used to discard 10% of the states as burn-in and to combine the trees from the two independent runs. A maximum clade credibility tree from the combined trees was built using TreeAnnotator v. 1.10.4 [59]. Finally, an ancestral reconstruction of Xf_PD_ was performed using discrete phylogeography analysis and setting the location of isolates (California versus southern US) as a trait in BEAST v. 1.10.

### (b) Phylodynamics

To determine the epidemic history of PD outbreaks, we estimated the effective population size of the major clades and subclades in the time-scaled phylogenies using Skygrowth [36]. This method reconstructs trends in epidemic growth and decline from time-scaled genealogies using a non-parametric autoregressive model on growth rate as a prior for effective population size. For both Taiwan and the southeastern US, we used the best-fitting phylogeny tree obtained with a GTR and HKY-strict clock model combined with the Bayesian skyline coalescent model (electronic supplementary material, table S1). The best-fitting time-scaled phylogenetic model for Xf_PD_ was used to estimate the epidemic history for the Californian clade. All tips not belonging to the Californian clade were removed from the tree, applying the *to drop* function in *ape*, except for isolate 14B1 from Georgia used as an outgroup. The basic reproduction number *R*_0_ for each outbreak was derived from the start of the epidemic (*x* = tMRCA) in the plots of reproduction *R*_e_ over time. Because the risk index of our epidemiological model gives a measure of the number of infected plants over time (see below), we tested a Spearman’s rank correlation ρ between the growth rate and the risk index using R v. 3.5.2 software.

### (c) Inoculation trials on rootstocks

We tested whether rootstocks differ in their susceptibility in relation to scions (grafted onto rootstocks). The main rootstocks currently used in Europe are the result of breeding efforts to find resistance to phylloxera among native American *Vitis* species in the late nineteenth century. Seven rootstock combinations were used in the inoculation tests (electronic supplementary material, table S2). Potted plants were distributed in rows of 12 plants along an insect-proof tunnel exposed to ambient temperature, watered daily to field capacity and fertilized fortnightly with a slow-release fertilizer. Two weeks before the onset of the inoculation test, leaf samples were tested for the presence of *Xf* by qPCR as previously described [28].

### (d) Isolates and inoculation

Two isolates of *Xf.* subsp. *fastidiosa* (ST1)—XLY 2055/17 and XYL2177/18—from grapevines on the island of Majorca (Spain), were used in the inoculation tests [28,63]. Isolates were grown on BYCE medium at 28°C for 7–10 days, following EPPO protocols [64]. Cells were harvested by scraping the colonies and suspending them in 1.5 ml Eppendorf tubes with 1 ml phosphate-buffered saline (PBS) solution until obtaining a turbid (10^8^–10^9^ cell ml^–1^) suspension. Plants were inoculated mechanically by pin-prick inoculation [17] with slight modifications. A 10 μl drop of the bacterial suspension was pipetted onto the leaf axil, which was punctured five times with an entomological needle. Eight to nine replicates per rootstock combination were inoculated with the bacterial suspension and 3–4 plants per rootstock with a drop of PBS as a control. Inoculation was repeated 2 weeks thereafter by piercing the next leaf axil above the one that was previously inoculated [65].

### (e) Disease scoring

The severity of the disease was quantified by counting the number of symptomatic leaves above the point of inoculation at 8 weeks post-inoculation (WPI) and then biweekly until the 16th week. A within-subject (repeated measures) factorial design was employed to evaluate the temporal differences in disease severity among different rootstocks. To compare the differences in susceptibility between American *Vitis* species (rootstocks) and *V. vinifera* varieties (rootstock–scion combinations), previously published data on disease scoring of rootstock–scion combinations were utilized [21]. The rootstock, cultivars–rootstock and time were treated as fixed factors and plant subjects as a random effect. Statistical analyses were performed using a generalized linear mixed models (GLMMs) and generalized linear model (GLM) with R v. 3.5.2 software. We used the *glmer* function in the R package lme4 [66] for fitting GLMMs for the estimation of disease severity in the inoculation assays. The response variable—disease severity—was modelled using a Poisson error distribution with a log-link function.

### (f) Epidemiological modelling

We employed a previously published climate-driven epidemiological model to analyse the epidemic risk for PD in Europe [32]. Briefly, the model is based on a standard Susceptible–Infected–Recovered (SIR) compartmental model of PD transmission from infected to susceptible plants, which relates the transmission and recovery rates together with the vector abundance to the basic reproductive number (*R*_0_) at the onset of an outbreak. To reflect the heterogeneous distribution of the vector *P. spumarius* in Europe, a spatial-dependent *R*_0_(*j*) is incorporated into the model. The epidemic risk maps were computed through simulation of the epidemiological model in which each grid cell was seeded with a single infected plant at (*t*_0_) = 1. The simulation ran from the initial to the final year in periods of 7 years using an annual time-step, computing the incidence. Cells from which the number of infected plants had vanished at the end of each period were seeded again. This process was repeated until the last period, in which no reseeding was performed to allow the system to relax. Finally, the risk index *r* was calculated from the final number of infected plants at grid cell *j*. The risk index *r*_*j*_(*T*) implicitly defines three differential risk zones in the maps: (i) non-risk zones where *r*_*j*_ ≤ −0.1, and the number of infected plants decreases exponentially; (ii) transition areas where −0.1 < *r*_*j*_ ≤ 0.1; and (iii) an epidemic risk-zone where *r*_*j*_ > 0.1 and PD can theoretically become established and produce an outbreak—where the number of infected plants increases exponentially. Full details on the model are available in previous works [32,42].

### (g) Climate data

Climate data between 1850 and 2023 from different sources were used to generate historical PD risk maps. Maximum and minimum daily temperature projections of each of the climate models were downloaded from the CMIP5 of the fifth phase of the experiment from 1850 to the present (https://cds.climate.copernicus.eu/cdsapp#!/dataset/projections-cmip5-daily-single-levels?tab=overview). Hourly temperature values were computed using a sinusoidal extrapolation relating maximum and minimum daily temperature to hourly temperatures [42]. Out of 16 climate models, we discarded those that reflected evident temporal and spatial inconsistencies in the risk map simulations with respect to the other models. Eventually, seven models, ‘CESM1-BGC’, ‘CSIRO-Mk3-6-0’, ‘NorESM1-M’, ‘ACCESS1-0’ and ‘CCSM4’, ‘IPSL-CM5A-MR’ and ‘ACCESS1-3’ were selected to average the infection probabilities of the US and European maps. The spatial resolution of all models was standardized to an average resolution of 1° using a first-order remapping climate data operator implemented by the Max-Plank Institute for Meteorology. In the PD risk simulations, we assumed that the population density and distribution of the insect vector *P. spumarius* in Europe did not vary during the period of study. Owing to the low resolution of the CMIP5 climate data, risk maps from 1950 to the present were constructed from ERA5-Land data [67] with a resolution of 0.1°. The data were downloaded in GRIB format with hourly temporal resolution and 0.1° spatial resolution. A Julia library [68] was developed using the GRIB.jl package [36] to compute the annual MGDD and Cold Degree Days (CDD) estimates. The risk of PD in Europe and the USA before 1950 was determined by evaluating the annual thermal anomalies and the risk of PD with the models simulated between 1950 and 2023 with the reanalysed ERA5-Land data. The data on thermal anomalies between 1850 and 2023 with respect to the 1901–2000 average (by cells) and with respect to the 1991–2020 average (coordinates) were obtained from the NOAA (https://www.ncei.noaa.gov/access/monitoring/climate-at-a-glance/global/time-series).

## Data Availability

Records of natural infections of wild American *Vitis* spp. were searched in the updated EFSA host database [45] and Google Scholar. Distribution records of native American *Vitis* spp. were downloaded from the United States Department of Agriculture [69]. The list of genomes used in the study is provided in electronic supplementary material, dataset S1 [70]. Files with data of annotated genomes, multiple sequence alignments, phylogenetic models built with Beauti and Beast output log, ops and tree files needed to reproduce the phylogenetic analyses are available in [71]. Hourly temperature data from 1950 to 2023 were taken from Copernicus ERA5-Land (namely the ‘2m temperature’ field). Maximum and minimum daily temperature data from CHELSA dataset downloaded to build movie 5 are available at Zenodo [72]. R scripts used for statistical analyses and plotting are available in [71]. A library built in Julia to analyse the data outputs of ERA5-Land in GRIB format is available at [71]. The simulation code and a small reproducible example are provided in [71] and [73].

## References

[1] Borkar SG. 2017 History of plant pathology. New York, NY: CRC Press.

[2] Brewer MT, Milgroom MG. 2010 Phylogeography and population structure of the grape powdery mildew fungus, Erysiphe necator, from diverse Vitis species. BMC Evol. Biol. **10**, 268. (10.1186/1471-2148-10-268) 20809968 PMC2941690

[3] Tello J, Mammerler R, Čajić M, Forneck A. 2019 Major outbreaks in the nineteenth century shaped grape Phylloxera contemporary genetic structure in Europe. Sci. Rep. **9**, 17540. (10.1038/s41598-019-54122-0) 31772235 PMC6879566

[4] Rouxel M *et al*. 2014 Geographic distribution of cryptic species of Plasmopara viticola causing downy mildew on wild and cultivated grape in Eastern North America. Phytopathology **104**, 692–701. (10.1094/PHYTO-08-13-0225-R) 24915427

[5] Hulme PE. 2009 Trade, transport and trouble: managing invasive species pathways in an era of globalization. J. Appl. Ecol. **46**, 10–18. (10.1111/j.1365-2664.2008.01600.x)

[6] Hopkins DL, Purcell AH. 2002 Xylella fastidiosa: cause of Pierce’s disease of grapevine and other emergent diseases. Plant Dis. **86**, 1056–1066. (10.1094/PDIS.2002.86.10.1056) 30818496

[7] Golino DA, Fuchs M, Al Rwahnih M, Farrar K, Schmidt A, Martelli GP. 2017 Regulatory aspects of grape viruses and virus diseases: certification, quarantine, and harmonization. In Grapevine viruses: molecular biology, diagnostics and management (eds B Meng, GP Martelli, DA Golino, M Fuchs), pp. 581–598. Cham, Switzerland: Springer International Publishing. (10.1007/978-3-319-57706-7_28)

[8] Janse JD, Obradovic A. 2010 Xylella fastidiosa: its biology, diagnosis, control and risks. J. Plant Pathol. **92**, 35–48 (doi:10.4454/jpp.v92i1sup.2504).

[9] Hoddle MS. 2004 The potential adventive geographic range of glassy-winged sharpshooter, Homalodisca coagulata and the grape pathogen Xylella fastidiosa: implications for California and other grape growing regions of the world. Crop Prot. **23**, 691–699. (10.1016/j.cropro.2003.11.017)

[10] Pierce NB. 1892 The California vine disease: a preliminary report of investigations. In USDA Division of Vegetable Pathology. Bulletin 2. Washington, DC: Government Printing Office.

[11] Stoner WN. 1952 A comparison between grape degeneration in Florida and Pierce’s disease in California. Florida Entomol. **35**, 62. (10.2307/3492278)

[12] Winkler AJ. 1949 Pierce’s disease investigations. Hilgardia **19**, 207–264. (10.3733/hilg.v19n07p207)

[13] Davis MJ, Purcell AH, Thomson SV. 1978 Pierce’s disease of grapevines: isolation of the causal bacterium. Science **199**, 75–77. (10.1126/science.199.4324.75) 17569487

[14] Hewitt WB. 1958 The probable home of Pierce’s disease virus. Am. J. Enol. Viticult. **9**, 94–98. (10.5344/ajev.1958.9.2.94)

[15] Schuenzel EL, Scally M, Stouthamer R, Nunney L. 2005 A multigene phylogenetic study of clonal diversity and divergence in North American strains of the plant pathogen Xylella fastidiosa. Appl. Environ. Microbiol. **71**, 3832–3839. (10.1128/AEM.71.7.3832-3839.2005) 16000795 PMC1169037

[16] Yuan X, Morano L, Bromley R, Spring-Pearson S, Stouthamer R, Nunney L. 2010 Multilocus sequence typing of Xylella fastidiosa causing Pierce’s disease and oleander leaf scorch in the United States. Phytopathology **100**, 601–611. (10.1094/PHYTO-100-6-0601) 20465416

[17] Almeida RPP, Purcell AH. 2003 Biological traits of Xylella fastidiosa strains from grapes and almonds. Appl. Environ. Microbiol. **69**, 7447–7452. (10.1128/AEM.69.12.7447-7452.2003) 14660397 PMC309917

[18] Aguilar-Granados A, Hernández-Macías B, Santiago-Martínez G, Ruiz-Medrano R, Kameyama-Kawabe L, Hinojosa-Moya J, Del Carmen Montes-Horcasitas M, Xoconostle-Cázares B. 2021 Genetic diversity of Xylella fastidiosa in Mexican vineyards. Plant Dis. **105**, 1490–1494. (10.1094/PDIS-09-20-1900-RE) 33780269

[19] Vanhove M, Retchless AC, Sicard A, Rieux A, Coletta-Filho HD, Fuente L, Stenger DC, Almeida RP. 2019 Genomic diversity and recombination among Xylella fastidiosa subspecies. Appl. Environ. Microbiol. **85**, e02972–18. (10.1128/AEM.02972-18) 31028021 PMC6581164

[20] Su CC, Chang CJ, Chang CM, Shih HT, Tzeng KC, Jan FJ, Kao CW, Deng WL. 2013 Pierce’s disease of grapevines in Taiwan: isolation, cultivation and pathogenicity of Xylella fastidiosa. J. Phytopathol. **161**, 389–396. (10.1111/jph.12075)

[21] Moralejo E *et al*. 2020 Phylogenetic inference enables reconstruction of a long-overlooked outbreak of almond leaf scorch disease (Xylella fastidiosa) in Europe. Commun. Biol. **3**, 560. (10.1038/s42003-020-01284-7) 33037293 PMC7547738

[22] Zecharia N *et al*. 2022 Xylella fastidiosa outbreak in Israel: population genetics, host range and temporal and spatial distribution analysis. Phytopathology **112**, 2296–2309. (10.1094/PHYTO-03-22-0105-R) 35778787

[23] Vanhove M, Sicard A, Ezennia J, Leviten N, Almeida RP. 2020 Population structure and adaptation of a bacterial pathogen in California grapevines. Environ. Microbiol. **22**, 2625–2638. (10.1111/1462-2920.14965) 32114707

[24] Castillo AI, Bojanini I, Chen H, Kandel PP, De La Fuente L, Almeida RPP. 2021 Allopatric plant pathogen population divergence following disease emergence. Appl. Environ. Microbiol. **87**, e02095-20. (10.1128/AEM.02095-20) 33483307 PMC8091610

[25] Nunney L, Yuan X, Bromley R, Hartung J, Montero-Astúa M, Moreira L, Ortiz B, Stouthamer R. 2010 Population genomic analysis of a bacterial plant pathogen: novel insight into the origin of Pierce’s disease of grapevine in the US. PLoS One **5**, e15488. (10.1371/journal.pone.0015488) 21103383 PMC2982844

[26] Saponari M, Boscia D, Nigro F, Martelli GP. 2013 Identification of DNA sequences related to xylella fastidiosa in oleander, almond and olive trees exhibiting leaf scorch symptoms in apulia (southern italy). J. Plant Pathol. **95**, 668. (10.4454/JPP.V95I3.035)

[27] Landa BB *et al*. 2020 Emergence of a plant pathogen in Europe associated with multiple intercontinental introductions. Appl. Environ. Microbiol. **86**, e01521–19. (10.1128/AEM.01521-1941) 31704683 PMC6974645

[28] Moralejo E *et al*. 2019 Insights into the epidemiology of Pierce’s disease in vineyards of Mallorca, Spain. Plant Pathol. **68**, 1458–1471. (10.1111/ppa.13076)

[29] Cornara D, Cavalieri V, Dongiovanni C, Altamura G, Palmisano F, Bosco D, Porcelli F, Almeida RPP, Saponari M. 2017 Transmission of Xylella fastidiosa by naturally infected Philaenus spumarius (Hemiptera, Aphrophoridae) to different host plants. J. Appl. Entomol. **141**, 80–87. (10.1111/jen.12365)

[30] Godefroid M, Cruaud A, Streito JC, Rasplus JY, Rossi JP. 2019 Xylella fastidiosa: climate suitability of European continent. Sci. Rep. **9**, 1–10. (10.1038/s41598-019-45365-y) 31222007 PMC6586794

[31] Schneider K, Werf W, Cendoya M, Mourits M, Navas-Cortés JA, Vicent A, Lansink AO. 2020 Impact of Xylella fastidiosa subspecies pauca in European olives. Proc. Natl Acad. Sci. USA **117**, 9250–9259. (10.1073/pnas.1912206117) 32284411 PMC7196823

[32] Giménez-Romero A, Galván J, Montesinos M, Bauzà J, Godefroid M, Fereres A, Ramasco JJ, Matías MA, Moralejo E. 2022 Global predictions for the risk of establishment of Pierce’s disease of grapevines. Commun. Biol. **5**, 1389. (10.1038/s42003-022-04358-w) 36539523 PMC9768138

[33] Viala P. 1889 Une mission viticole en amérique. Paris, France: C. Coulet.

[34] Hewitt WB. 1953 Virus diseases of grapevines. Yearbook Agr. US Dept. Agric. 744–753.

[35] Selander RK, Musser JM, Caugant DA, Gilmour MN, Whittam TS. 1987 Population genetics of pathogenic bacteria. Microb. Pathog. **3**, 1–7. (10.1016/0882-4010(87)90032-5) 3332801

[36] Volz EM, Didelot X. 2018 Modeling the growth and decline of pathogen effective population size provides insight into epidemic dynamics and drivers of antimicrobial resistance. Syst. Biol. **67**, 719–728. (10.1093/sysbio/syy007) 29432602 PMC6005154

[37] Mircetich SM, Lowe SK, Moller WJ, Nyland G. 1976 Etiology of almond leaf scorch disease and transmission of the causal agent. Phytopathology **66**, 17. (10.1094/Phyto-66-17)

[38] Sorensen CW, Smith EH, Smith J, Carton Y. 2008 Charles V. Riley, France, and Phylloxera. Am. Entomol. **54**, 134–149. (10.1093/ae/54.3.134)

[39] Gale G. 2002 Saving the vine from phylloxera: a never ending battle. In Wine: a scientific exploration. London, UK: CRC Press. See https://www.taylorfrancis.com/chapters/edit/10.4324/9780203361382-9/saving-vine-phylloxera-never-ending-battle-gale?context=ubx&refId=545d1412-5aad-46bb-aed6-b88695cece56.

[40] Loarie SR, Duffy PB, Hamilton H, Asner GP, Field CB, Ackerly DD. 2009 The velocity of climate change. Nature **462**, 1052–1055. (10.1038/nature08649) 20033047

[41] Riaz S, Tenscher AC, Heinitz CC, Huerta-Acosta KG, Walker MA. 2020 Genetic analysis reveals an east–west divide within North American Vitis species that mirrors their resistance to Pierce’s disease. PLoS One **15**, e0243445. (10.1371/journal.pone.0243445) 33338052 PMC7748146

[42] Giménez-Romero À, Iturbide M, Moralejo E, Gutiérrez JM, Matías MA. 2024 Global warming significantly increases the risk of Pierce’s disease epidemics in European vineyards. Sci. Rep. **14**, 9648. (10.1038/s41598-024-59947-y) 38671045 PMC11576866

[43] Lemey P, Rambaut A, Drummond AJ, Suchard MA. 2009 Bayesian phylogeography finds its roots. PLoS Comput. Biol. **5**, e1000520. (10.1371/journal.pcbi.1000520) 19779555 PMC2740835

[44] Bush & Son & Meissner. 1883 Illustrated descriptive catalogue of american grape vines: a grape growers’ manual. St. Louis, MO: RP Studley & Company printers.

[45] Delbianco A, Gibin D, Pasinato L, Boscia D, Morelli M, European Food Safety Authority (EFSA). 2023 Update of the Xylella spp. host plant database – systematic literature search up to 30 June 2022. EFSA J. **21**, e07726. (10.2903/j.efsa.2023.7726) 36628332 PMC9827234

[46] Morano LD, Bextine BR, Garcia DA, Maddox SV, Gunawan S, Vitovsky NJ, Black MC. 2008 Initial genetic analysis of Xylella fastidiosa in Texas. Curr. Microbiol. **56**, 346–351. (10.1007/s00284-007-9088-2) 18172717

[47] Stover E, Riaz S, Walker MA. 2008 PCR screening for Xylella fastidiosa in grape genebank accessions collected in the Southeastern United States . Am. J. Enol. Vitic. **59**, 437–439. (10.5344/ajev.2008.59.4.437)

[48] Richter M, Rosselló-Móra R, Oliver Glöckner F, Peplies J. 2016 JSpeciesWS: a web server for prokaryotic species circumscription based on pairwise genome comparison. Bioinformatics **32**, 929–931. (10.1093/bioinformatics/btv681) 26576653 PMC5939971

[49] Bankevich A *et al*. 2012 SPAdes: a new genome assembly algorithm and its applications to single-cell sequencing. J. Comput. Biol. **19**, 455–477. (10.1089/cmb.2012.0021) 22506599 PMC3342519

[50] Gurevich A, Saveliev V, Vyahhi N, Tesler G. 2013 QUAST: quality assessment tool for genome assemblies. Bioinformatics **29**, 1072–1075. (10.1093/bioinformatics/btt086) 23422339 PMC3624806

[51] Darling AE, Mau B, Perna NT. 2010 Progressivemauve: multiple genome alignment with gene gain, loss and rearrangement. PLoS One **5**, e11147. (10.1371/journal.pone.0011147) 20593022 PMC2892488

[52] Seemann T. 2014 Prokka: rapid prokaryotic genome annotation. Bioinformatics **30**, 2068–2069. (10.1093/bioinformatics/btu153) 24642063

[53] Page AJ *et al*. 2015 Roary: rapid large-scale prokaryote pan genome analysis. Bioinformatics **31**, 3691–3693. (10.1093/bioinformatics/btv421) 26198102 PMC4817141

[54] Stamatakis A. 2015 Using RAxML to infer phylogenies. Curr. Protoc. Bioinformatics **51**, 6–14. (10.1002/0471250953.bi0614s51) 26334924

[55] Croucher NJ, Page AJ, Connor TR, Delaney AJ, Keane JA, Bentley SD, Parkhill J, Harris SR. 2015 Rapid phylogenetic analysis of large samples of recombinant bacterial whole genome sequences using Gubbins. Nucleic Acids Res. **43**, e15. (10.1093/nar/gku1196) 25414349 PMC4330336

[56] Nguyen LT, Schmidt HA, von Haeseler A, Minh BQ. 2015 IQ-TREE: a fast and effective stochastic algorithm for estimating maximum-likelihood phylogenies. Mol. Biol. Evol. **32**, 268–274. (10.1093/molbev/msu300) 25371430 PMC4271533

[57] Kalyaanamoorthy S, Minh BQ, Wong TK, Von Haeseler A, Jermiin LS. 2017 ModelFinder: fast model selection for accurate phylogenetic estimates. Nat. Methods. **14**, 587–589. (10.1038/nmeth.4285) 28481363 PMC5453245

[58] Yu G, Smith DK, Zhu H, Guan Y, Lam TY. 2017 ggtree: an R package for visualization and annotation of phylogenetic trees with their covariates and other associated data. Methods Ecol. Evol. **8**, 28–36. (10.1111/2041-210X.12628)

[59] Suchard MA, Lemey P, Beales G, Ayres DL, Drummond AJ, Rambaut A. 2018 Bayesian phylogenetic and phylodynamic data integration using BEAST 1.10. Virus Evol. **4**, vey016. (10.1093/ve/vey016) 29942656 PMC6007674

[60] Rambaut A, Lam TT, Max Carvalho L, Pybus OG. 2016 Exploring the temporal structure of heterochronous sequences using TempEst (formerly Path-O-Gen). Virus Evol. **2**, vew007. (10.1093/ve/vew007) 27774300 PMC4989882

[61] Rieux A, Khatchikian CE. 2017 TipDatingBeast: an R package to assist the implementation of phylogenetic tip‐dating tests using BEAST. Mol. Ecol. Resour. **17**, 608–613. (10.1111/1755-0998.12603) 27717245

[62] Rambaut A, Drummond AJ, Xie D, Baele G, Suchard MA. 2018 Posterior summarization in Bayesian phylogenetics using Tracer 1.7. Syst. Biol. **67**, 901–904. (10.1093/sysbio/syy032) 29718447 PMC6101584

[63] Gomila M, Moralejo E, Busquets A, Segui G, Olmo D, Nieto A, Juan A, Lalucat J. 2019 Draft genome resources of two strains of Xylella fastidiosa XYL1732/17 and XYL2055/17 isolated from Mallorca vineyards. Phytopathology **109**, 222–224. (10.1094/PHYTO-08-18-0298-A) 30570447

[64] EPPO. 2018 PM 7/24 (3) Xylella fastidiosa. EPPO. Bull. **48**, 175–218. (10.1111/epp.12469)

[65] Wallis CM, Wallingford AK, Chen J. 2013 Grapevine rootstock effects on scion sap phenolic levels, resistance to Xylella fastidiosa infection, and progression of Pierce’s disease. Front. Plant Sci. **4**, 502. (10.3389/fpls.2013.00502) 24376452 PMC3860182

[66] Bates D, Mächler M, Bolker B, Walker S. 2014 Fitting linear mixed-effects models using lme4. arXiv 1406.5823. (10.48550/arXiv.1406.5823)

[67] Hersbach H *et al*. 2020 The ERA5 global reanalysis. Quart. J. Royal Meteoro. Soc. **146**, 1999–2049. (10.1002/qj.3803)

[68] Bezanson J, Edelman A, Karpinski S, Shah VB. 2017 Julia: a fresh approach to numerical computing. SIAM Rev. **59**, 65–98. (10.1137/14100067)

[69] United States Department of Agriculture (USDA), Natural Resources Conservation Service (NRCS),. 2024 The PLANTS Database, National Plant Data Team, Greensboro, NC USA. See http://plants.usda.gov.

[70] Moralejo E, Giménez-Romero À, Matías MA. 2024 Data from: Linking intercontinental biogeographic events to decipher how European vineyards escaped Pierce’s disease. Figshare. (10.6084/m9.figshare.c.7468072)39353554

[71] Moralejo E, Giménez-Romero À, Matías MA. 2024 Data for: Linking intercontinental biogeographic events to decipher how European vineyards escaped Pierce’s disease. Dryad Digital Repository. (10.5061/dryad.s4mw6m9fz)39353554

[72] Giménez-Romero À, Moralejo E, Matías MA. 2024 High-resolution climate data reveals increased risk of Pierce’s Disease for grapevines worldwide. Zenodo. (10.5281/zenodo.10579689) PMC12379226 40855074

[73] Moralejo E, Giménez-Romero À, Matías MA. 2024 Linking intercontinental biogeographic events to decipher how European vineyards escaped Pierce’s disease. Zenodo. (10.5281/zenodo.13227383)39353554

